# Re-thinking cell cycle regulators: the cross-talk with metabolism

**DOI:** 10.3389/fonc.2013.00004

**Published:** 2013-01-25

**Authors:** Lluis Fajas

**Affiliations:** Department of Physiology, Université de LausanneLausanne, Switzerland

**Keywords:** cell cycle, metabolism, mitochondria, cdk4, E2F1 transcription factor

## Abstract

Analysis of genetically engineered mice deficient in cell cycle regulators, including E2F1, cdk4, and pRB, showed that the major phenotypes are metabolic perturbations. These key cell cycle regulators contribute to lipid synthesis, glucose production, insulin secretion, and glycolytic metabolism. It has been shown that deregulation of these pathways can lead to metabolic perturbations and related metabolic diseases, such as obesity and type II diabetes. The cyclin–cdk–Rb–E2F1 pathway regulates adipogenesis in addition to its well-described roles in cell cycle regulation and cancer. It was also shown that E2F1 directly participates in the regulation of pancreatic growth and function. Similarly, cyclin D3, cdk4, and cdk9 are also adipogenic factors with strong effects on whole organism metabolism. These examples support the emerging notion that cell cycle regulatory proteins also modulate metabolic processes. These cell cycle regulators are activated by insulin and glucose, even in non-proliferating cells. Most importantly, these cell cycle regulators trigger the adaptive metabolic switch that normal and cancer cells require in order to proliferate. These changes include increased lipid synthesis, decreased oxidative metabolism, and increased glycolytic metabolism. In summary, these factors are essential regulators of anabolic biosynthetic processes, blocking at the same time oxidative and catabolic pathways, which is reminiscent of cancer cell metabolism.

## INTRODUCTION

**FIGURE 1:**
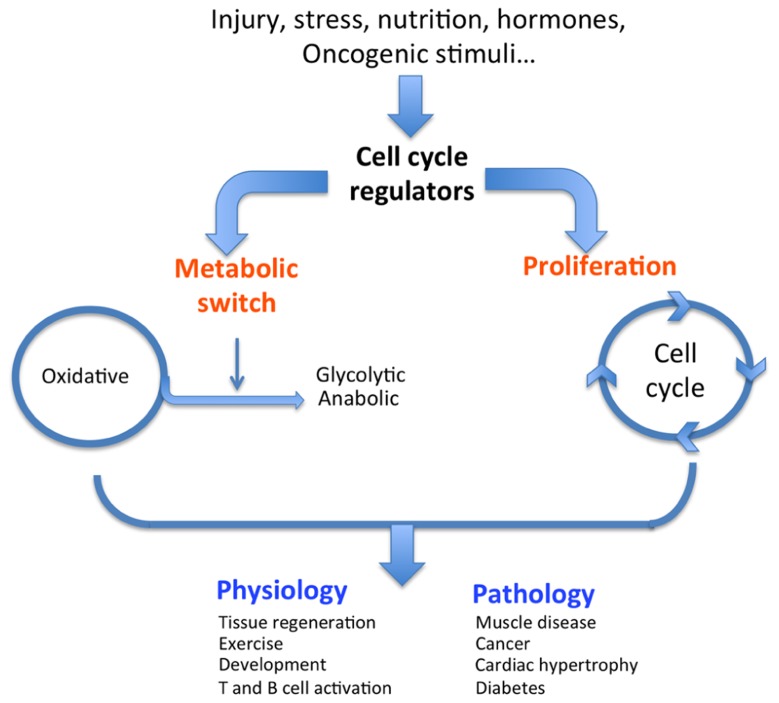
**Dual role of cell cycle regulators**. In response to external stimuli such as oncogenes, nutrition, or hormones, cell cycle regulators trigger both a classical proliferative burst and an adapted metabolic response (switch from oxidative to glycolytic), ultimately resulting in the physiological or pathological condition.

Most physiological and pathological changes in cellular functions are accompanied by an adapted metabolic switch. A regulated cascade of molecular events senses external conditions and triggers an adapted specific metabolic pathway. Each cell function, such as proliferation, survival, growth, and senescence, requires a specific adaptive metabolic response. Intermediary metabolism must be coupled to the needs of the cell (e.g., growth, proliferation, and function); in other words, intermediary metabolism must be coupled to either biosynthetic or oxidative metabolism. Changes in cellular status that require metabolic adaptation can be of physiological or pathological origin (**Figure 1**). As a general rule, cell proliferation and clonal expansion, as observed during tissue regeneration or developmental processes, create a strong demand for carbon sources (glucose) for the synthesis of membranes, organelles, and biomass. Glucose is also the source of energy and redox equivalents. These needs are fully satisfied by anaerobic glycolysis and anabolic processes such as protein, nucleic acid, and lipid biosynthesis. The preferential use of aerobic glycolysis offers several advantages to highly proliferating cells, concerning both bioenergetics and biosynthetic requirements. First, it allows for the use of the most abundant extracellular nutrient, which is glucose. Second, the flux of adenosine triphosphate (ATP) can exceed that produced during oxidative phosphorylation (OXPHOS). Third, enzymes involved in lipid biosynthesis have been shown to support cancer cell proliferation (Bauer et al., 2005). This supports the hypothesis that one of the critical roles of aerobic glycolysis is to provide essential metabolic intermediates for the biosynthesis of macromolecules (lipids, proteins, and nucleic acids) to support increased proliferation.

Physiological conditions that are typically glycolytic include vigorous anaerobic exercise. Under these conditions, high energy levels are required to rapidly provide ATP for muscle contraction (Jensen and Richter, 2012). Similarly, pluripotent stem cells present in adult tissues or during embryonic development rely heavily on glycolysis for self-renewal to maintain pluripotency (Zhang et al., 2011). Another physiological condition illustrating a glycolytic metabolic switch is T cell activation. During this process, T cells undergo a metabolic reprograming from oxidative metabolism in naïve cells to a glycolytic pathway in activated cells (Wang et al., 2011). These are normal physiological conditions that require changes in metabolism, but cells and tissues adapt their metabolic pathways under pathological conditions as well. A metabolic switch is also required to support the shift from normal to pathological cell or tissue function. Many pathologies, including diabetes, obesity, muscle disease, cardiac hypertrophy, and cancer, can be identified by specific metabolic changes. For example, hypertrophied hearts are characterized by increased glycolysis and decreased oxidative metabolism compared to normal hearts (Allard et al., 1994; Kolwicz and Tian, 2011). Strikingly, skeletal muscle of the mouse model and human patients of Duchene’s muscular dystrophy exhibit decreased oxidative metabolism (Kuznetsov et al., 1998; Chen et al., 2000). Finally, and most relevant for this review, we will discuss how cancer cells switch their metabolism and what molecular mechanisms are involved.

Changes in cancer cellular metabolism have been thoroughly reviewed elsewhere. It is well established that the process of cancer development and progression involves major alterations in cellular metabolism. Similarly to physiological conditions that demand large amounts of readily available energy, cancer cells are highly glycolytic. One of the first biochemical hallmarks of cancer cells to be identified was an alteration in metabolism. Early in the last century, Otto Warburg (1928) observed that tumors have a higher rate of glucose metabolism than normal tissues (Warburg, 1930, 1956a,b). Since this first observation, the “aerobic” glycolysis switch has been detected in many tumor types, and cumulating studies on various proliferating cells have demonstrated evidence of a global metabolic change that takes place during cancer progression. Most tumors are characterized by higher rates of glycolysis, lactate production, and macromolecule and lipid biosynthesis (Kroemer and Pouyssegur, 2008; Vander Heiden et al., 2009). During aerobic glycolysis, glucose is converted to pyruvate, and the final product of this reaction is lactate, which is exported out of the cell. Lactic acidosis is a typical complication in cancer patients (Dhup et al., 2012). Although de novo fatty acid (FA) synthesis is very active during embryogenesis, most adult normal cells and tissues, even those with high cellular turnover, preferentially use circulating FA for the synthesis of new structural lipids. In contrast, various tumors and their precursor lesions undergo exacerbated endogenous FA biosynthesis irrespective of the levels of extracellular lipids (Medes et al., 1953; Kuhajda, 2000; Pizer et al., 2000).

The molecular mechanisms underlying the metabolic switch observed in cancer cells are poorly understood. In addition to triggering signaling cascades involved in proliferation and survival, oncogenes (e.g., as ras, wnt, and AKT), cell cycle regulators (e.g., the cdk4–E2F1 axis), and some viral gene products also trigger metabolic changes. Participation of these oncogenes in the inhibition of oxidative metabolism and increased glycolysis, which are among the hallmarks of cancer, is well documented (Jones et al., 2005; Shaw, 2006; Manning and Cantley, 2007; Wise et al., 2008; Guo et al., 2011; Garcia-Cao et al., 2012). Coupling cellular function with the adapted metabolic response has to be controlled by the same signaling network. Cell cycle regulators, notably the cdk4–pRB–E2F1 axis, are the crucial factors in the control of cell proliferation. It is therefore not surprising that they are also key factors in metabolic control. Herein were view the relative participation of cell cycle regulators in the regulation of cell proliferation, transformation, and metabolic control.

## THE CLASSICAL ROLE OF CELL CYCLE REGULATORS: PROLIFERATION AND SURVIVAL

Cyclins, cyclin-dependent kinases (cdks), transcription factor E2F1, and retinoblastoma protein pRB are major regulators of cell growth, development, and proliferation and are good candidates as sensors of external signals that require a particular adapted metabolic response. The most studied role of these factors is the regulation of cell cycle progression in proliferating cells. Entry into S-phase of the cell cycle depends on the activation of the G_1_ cyclins/cdks and the pRB–E2F pathway that controls the G_1_/S transition of the cell cycle. Cdks are serine/threonine kinases that work in complexes with different types of cyclins to phosphorylate the retinoblastoma family of tumor suppressor proteins (pRB) mediating the commitment of the cells to enter the cell cycle in response to external stimuli (reviewed in Ortega et al., 2002). E2F transcription factors are the effectors of this pathway, and they control the expression of genes involved in cell cycle progression, apoptosis, and DNA synthesis (for review, see Attwooll et al., 2004). E2Fs regulate transcription through heterodimerization with members of the DP family (DP-1 and DP-2; Dyson, 1998). When bound to DNA, this heterodimeric complex exists as free E2F/DP or forms a larger complex that contains a member of the retinoblastoma protein family (pRB, p107, p130). E2F complexes can activate (free heterodimers) or repress (large complexes) the transcription of E2F-responsive genes. E2F activity is commonly increased in many human cancers, including glioblastoma and lung, ovarian, breast, stomach, and colon cancers (Chen et al., 2009), and much evidence supports an oncogenic role for E2F1–3. It is clear that the increased expression of E2Fs contributes to the uncontrolled proliferation of cancer cells. We will discuss in this review the role of the cdk–pRB–E2F axis as part of the control pathway of the metabolic adaptive response triggered by growth factors.

## THE NEW ROLE OF CELL CYCLE REGULATORS: METABOLIC CONTROL

There is increasing evidence for a cdk4–E2F1–pRB-specific effect in metabolism. This includes a recent observation implicating pRB in the control of oxidative metabolism in adipose tissue (Dali-Youcef et al., 2007). In addition, we previously demonstrated that cell cycle regulators participate in lipid metabolism. We showed that E2Fs regulate adipogenesis by modulating the expression of the nuclear receptor PPARγ, which is a master regulator of adipogenesis (Fajas et al., 2002b). Similarly, we documented the adipogenic role of cyclin D3 (Sarruf et al., 2005), cdk4 (Abella et al., 2005), and cdk9 (Iankova et al., 2006) through their positive regulation of PPARγ activity. We also found that PPARγ activity and adipocyte differentiation are repressed by RB through the recruitment of HDAC3 (Fajas et al., 2002a).

Analysis of genetically engineered mice deficient for cell cycle regulators, notably E2F1, cdk4, cyclin D3, p21, p19, and pRB, show that the major phenotypes are metabolic perturbations. It is shown that some of these cell cycle regulators are crucial factors in metabolic control. Cdk4^-/-^ and E2F1^-/-^ mice have altered glucose homeostasis and impaired mitochondrial function, reflecting profound metabolic changes. In contrast, cdk4R24C mutant mice with highly active cdk4 (almost constitutive) are obese (Aguilar and Fajas, 2010). Interestingly, these metabolic changes are also part of the metabolic switch observed in cancer, as discussed below.

**FIGURE 2:**
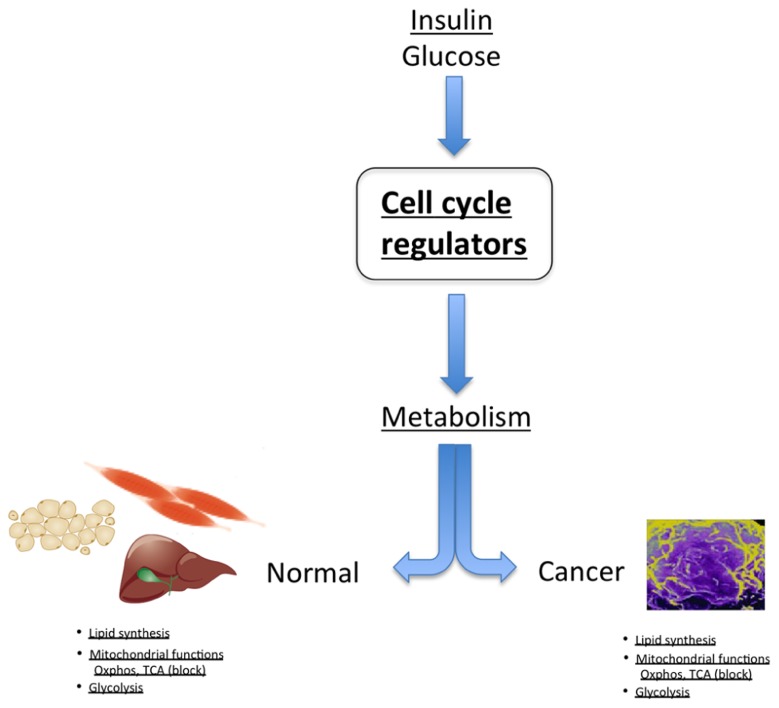
**In normal tissue, cell cycle regulators control metabolic processes, such as regulation of lipid synthesis, glycolysis, and modulation of mitochondrial activity**. Strikingly, these are the same metabolic pathways that are deregulated in cancer cells.

An example of the dual proliferation-metabolism regulatory network is the regulation of cell physiology by insulin and glucose. These factors stimulate proliferation of some cell types, whereas they trigger a metabolic response in other cells, such as pancreatic β-cells. Interestingly, cdk4 is activated by insulin in pancreatic β-cells. Indeed, the cdk4–Rb–E2F network is a sensor of the nutritional and energetic status of the cell, in particular in pancreatic β-cells, where E2F1 regulates the expression of the Kir6.2 gene, facilitating insulin secretion in these cells (Annicotte et al., 2009). The cdk4–Rb–E2F pathway is also a negative regulator of energy expenditure, through repression of mitochondrial oxidative metabolism (Blanchet et al., 2011). Further support for this hypothesis comes from genome-wide studies showing that a cohort of genes involved in mitochondrial function are E2F targets, indicating a potential role for E2Fs in linking the metabolic state of the cell to cell cycle status (Cam et al., 2004). E2F1 transcriptional activation of the pyruvate dehydrogenase kinase 4 (PDK4) gene, a key nutrient sensor that is constitutively expressed in diabetes, was also demonstrated to regulate glucose homeostasis in muscle through inhibition of glucose oxidation (Hsieh et al., 2008). It was shown that inactivation of pRB by the oncogenic E1A adenoviral protein also triggers PDK4 activity (Hsieh et al., 2008). Increased E2F1 activity or inactivation of pRB therefore maintains high PDK4 expression and constitutively suppresses glucose oxidation, such as observed in cancer. Furthermore, E2F1 stimulates glycolytic flux through regulation of the expression of the phosphofructokinase 2 enzyme (Darville et al., 1995). In summary, these factors are essential regulators of anabolic biosynthetic processes, blocking oxidative and catabolic pathways, which is reminiscent of cancer cell metabolism (**Figure 2**).

E2F and pRB activities are regulated by cyclin/cdk complexes to facilitate cell cycle entry and progression. It is therefore expected that in the control of metabolism these holoenzymes also regulate metabolism. This is demonstrated by the analysis of cyclin D-deficient mice that show marked metabolic phenotypes, first observed in pancreas physiology. For instance, cyclin D2^-/-^ mice showed deregulated glucose homeostasis. This was the result of decreased postnatal β-cell mass, glucose intolerance, and diabetes (Georgia and Bhushan, 2004). Although cyclin D1^+/-^ mice were normal, life-threatening diabetes developed in 3-month-old cyclin D1^+/-^D2^-/-^ mice as β-cell mass decreased after birth (Kushner et al., 2005). The conclusion from these studies was that cyclins D2 and D1 were essential for β-cell expansion in adult mice. More extensive analysis indicated that, in a glucose-stimulated insulin secretion experiment, cyclin D2^-/-^ mice demonstrated fasting insulin levels similar to cyclin D2^+/+^ mice. Upon glucose stimulation, cyclin D2^-/-^ mice did not increase insulin levels, whereas cyclin D2^+/+^ mice doubled their insulin secretion (Georgia and Bhushan, 2004). This strongly suggests that, in addition to the control of β-cell mass, cyclin D2 also participates in the control of β-cell function. This is fully consistent with the function of E2F1 in these cells (Fajas et al., 2004; Annicotte et al., 2009).

Participation of cyclins in metabolism in not limited to the control of pancreatic function. Cyclin D3-deficient mice are protected from diet-induced obesity, exhibit reduced adipocyte size, and exhibit increased sensitivity to insulin (Sarruf et al., 2005). Cyclin D3 regulates adipose tissue mass through direct interaction with PPARγ, the master regulator of adipogenesis. Another example of metabolic control by cyclins includes the negative regulation of oxidative metabolism by cyclin D1. Transgenic mice expressing antisense cyclin D1 in the mammary gland of ErB2 mice showed increased expression of genes that enhance oxidative glycolysis, lipogenesis, and mitochondrial function (Sakamaki et al., 2006). At odds with the metabolic cancer phenotype that shows increased lipid synthesis is the observation that cyclin D1 inhibits lipogenesis in the liver in response to glucose stimulation. It was shown that cyclin D1 inhibits the activity of the carbohydrate response element-binding protein (ChREBP; Hanse et al., 2012). In contrast, it was shown in *C. elegans* that the cyclin D/cdk complex regulates the expression of biosynthetic genes (Korzelius et al., 2011). This effect may be limited to a particular physiological condition in the liver and probably does not reflect pathological conditions, such as observed during carcinogenesis. Finally, in support of a metabolic function of the cyclin D/cdk4 holoenzyme in metabolism is the finding that some specific polymorphisms in the cdk4 gene could contribute to type II diabetes-associated obesity (Meenakshisundaram and Gragnoli, 2009a). Strikingly, the same cdk4 IVS4-nt40 AA genotype is significantly associated with cancer in obese patients (Meenakshisundaram and Gragnoli, 2009b). In summary, cyclin Ds/cdk4 regulates both cell growth and metabolism, thus integrating both processes in cellular function in normal and in transformed cells.

Other members of the cdk family also contribute to metabolic control, such as cdk5. First, cdk5 is activated by glucose and insulin in pancreatic β-cells (Lilja et al., 2001; Ubeda et al., 2004) and adipocytes (Okada et al., 2008), respectively. Second, cdk5 participates in the control of insulin secretion in β-cells (Lilja et al., 2001). Third, cdk5 activity modulates the expression of a particular cluster of PPARγ genes in adipocytes, such as adiponectin, leptin, and adipsin (Choi et al., 2010). Finally, a common polymorphism of the cdk5 regulatory unit CDKAL1 has been associated with type II diabetes (Perry and Frayling, 2008). Similar to other members of the cdk family, cdk5 also regulates cancer cell growth and migration in glioblastoma (Liu et al., 2008), prostate cancer (Strock et al., 2006), and pancreatic cancer (Feldmann et al., 2010), among others. This further supports the hypothesis that the control of metabolic processes and cancer progression is not limited to a single pathway but rather is a general and common way of dual regulatory pathways.

The activities and functions of cyclin/cdk complexes are regulated by the cdk inhibitors (CKIs). In line with our arguments, we should also expect a metabolic function for these proteins. CKIs comprise two families. The first family includes the INK4 proteins (for inhibitors of cdk4), which specifically bind and inhibit the catalytic subunits of cdk4 and cdk6. The INK4 family includes the four members p16INK4a, p15INK4b, p18INK4c, and p19INK4d, and another unrelated protein designated as p19ARF. The second family of CKIs is the Cip/Kip, which has a broad inhibitive function, including inhibiting the activities of cyclins and CDKs. This family includes p21Cip1, p27Kip1, and p57Kip2. Disruption of CKI genes in the mouse has not revealed profound cell cycle abnormalities, but does result in specific tumor and metabolic phenotypes. Mice lacking p18INK4c are larger than wild-type mice, a phenotype that varies with genetic background (Franklin et al., 1998; Latres et al., 2000). This is consistent with the role of p18INK4c as a cdk4 inhibitor, since cdk4-deficient mice are small. Similarly, disruption of p27Kip1 induces enhanced growth of mice (Kiyokawa et al., 1996). Interestingly, it was demonstrated that p27 and p21 are important regulators of adipogenesis, and loss of either of these CKIs in genetically modified mouse models induces adipocyte hyperplasia (Naaz et al., 2004). Furthermore, combined deletion of p27 and p21 induces an increase in adipocyte number, fat pad weights, and obesity in the double knockout mice. Double knockout mice (p21^-/-^; p27^-/-^) developed hypercholesterolemia, glucose intolerance, and insulin insensitivity, which are metabolic adaptations of obesity (Naaz et al., 2004). Genetic data fully support the participation of CKI genes in metabolic pathways. The CDKN2A/B locus is associated with type II diabetes in several studies (Saxena et al., 2007; Scott et al., 2007). The exact molecular mechanisms underlying this association, however, are far from being elucidated.

Interestingly, mutations and polymorphisms in this locus are also found in cancer, which reinforces the hypothesis that cell cycle regulators play a dual role, regulating metabolic processes in normal cells and regulating both proliferation and metabolism in cancer cells (Hammerman et al., 2012; Sherr, 2012).

## CONCLUSION

We have reviewed here studies showing that cell cycle regulators control metabolic processes such as lipid synthesis, glycolysis, and mitochondrial function, all of them being involved in the metabolic switch required for cancer development and progression. This dual regulation, proliferation-metabolism, is a key event for the successful viability of transformed cells. Interventions directed at uncoupling the metabolic response from transformation signaling should impair cancer growth.

## Conflict of Interest Statement

The author declares that the research was conducted in the absence of any commercial or financial relationships that could be construed as a potential conflict of interest.
